# The effect of neural pre-stimulus oscillations on post-stimulus somatosensory event-related potentials in disorders of consciousness

**DOI:** 10.3389/fnins.2023.1179228

**Published:** 2023-06-09

**Authors:** Laura Lindenbaum, Inga Steppacher, Alexandra Mehlmann, Johanna Maria Kissler

**Affiliations:** ^1^Department of Psychology, Bielefeld University, Bielefeld, Germany; ^2^Center for Cognitive Interaction Technology (CITEC), Bielefeld University, Bielefeld, Germany

**Keywords:** disorder of consciousness, EEG frequency bands, EEG, somatosensory event-related potentials, unresponsive wakefulness syndrome, minimally conscious state

## Abstract

Brain activity of people in a disorder of consciousness (DoC) is diffuse and different from healthy people. In order to get a better understanding of their cognitive processes and functions, electroencephalographic activity has often been examined in patients with DoC, including detection of event-related potentials (ERPs) and spectral power analysis. However, the relationship between pre-stimulus oscillations and post-stimulus ERPs has rarely been explored in DoC, although it is known from healthy participants that pre-stimulus oscillations predispose subsequent stimulus detection. Here, we examine to what extent pre-stimulus electroencephalography band power in DoC relates to post-stimulus ERPs in a similar way as previously documented in healthy people. 14 DoC patients in an unresponsive wakefulness syndrome (UWS, *N* = 2) or a minimally conscious state (MCS, *N* = 12) participated in this study. In an active oddball paradigm patients received vibrotactile stimuli. Significant post-stimulus differences between brain responses to deviant and standard stimulation could be found in six MCS patients (42.86%). Regarding relative pre-stimulus frequency bands, delta oscillations predominated in most patients, followed by theta and alpha, although two patients showed a relatively normal power spectrum. The statistical analysis of the relationship between pre-stimulus power and post-stimulus event-related brain response showed multiple significant correlations in five out of the six patients. Individual results sometimes showed similar correlation patterns as in healthy subjects primarily between the relative pre-stimulus alpha power and post-stimulus variables in later time-intervals. However, opposite effects were also found, indicating high inter-individual variability in DoC patients´ functional brain activity. Future studies should determine on an individual level to what extent the relationship between pre- and post-stimulus brain activity could relate to the course of the disorder.

## Introduction

Millions of people worldwide suffer severe brain injury each year and fall into coma or are put into a medically induced coma. Most of these patients awake from the coma and recover, some of them have consequential damage after awakening and some do not survive. Among the consequential damages resulting from severe brain injuries are disorders of consciousness (DoC), which can be divided into different stages such as the minimally conscious state (MCS; Giacino et al., 2002) and the unresponsive wakefulness syndrome (UWS), formerly known as vegetative state (Laureys et al., 2010). Because of reduced behavioral responses in both conditions (Giacino and Kalmar, 1997), it is challenging to make a correct diagnosis to differentiate between UWS and MCS, but these states are distinguishable by circadian rhythms and the presence of conscious awareness (Giacino and Kalmar, 1997; Giacino et al., 2002). In UWS, patients do not establish genuine sleep-wakefulness cycles, they show altered sleep patterns with a disturbed circulation of sleep over the day and night (Mertel et al., 2020). They also lost their speech production and show no signs of awareness of the self- or the environment (Jennett and Plum, 1972). In MCS, most patients have preserved sleep-wakefulness cycles and they appear to understand single words or short phrases and show minimal but definite evidence of self-awareness and/or awareness of the environment (Giacino et al., 2002, 2014).

Minimally conscious state can be further subdivided into MCS + and MCS-depending on the complexity of the behavioral responses of the patient where MCS + is diagnosed for patients with high-level (e.g., command following, intelligible verbalizations or gestural or verbal yes/no responses) and MCS- for patients with low-level responses (e.g., orientation of noxious stimuli, pursuit eye movements that occur in direct response to moving or salient stimuli, movements or affective behaviors that occur appropriately in relation to relevant environmental stimuli; Bruno et al., 2011). The prognosis for recovery from both, the MCS and UWS state can vary widely. They can become a permanent condition without changes of the cognitive status of the patient for years until ultimate death, but patients can also make progress towards recovery (Steppacher et al., 2016, 2020), whereby patients in MCS, and those who have reached MCS faster after injury and coma, seem to have a better chance to do so (Giacino and Kalmar, 1997; Giacino and Kalmar, 2005; Voss et al., 2006; Luauté et al., 2010; Hirschberg and Giacino, 2011).

Neuroimaging research using multiple methods such as functional magnetic resonance imaging (fMRI), positron emission tomography (PET), electroencephalography (EEG) and magnetoencephalography (MEG) has been performed to study the brain activity in patients with DoC in attempts to get a better understanding of their cognitive processes and functions. These studies reveal that sometimes patients that are behaviorally diagnosed as UWS have brain activity that is indicative of higher-order cognitive processes and even resembles what is found in healthy people (e.g., Owen et al., 2006; Cruse et al., 2011). The outcomes of such studies may help to develop prognostic tools but also new treatment strategies and techniques, such as brain–computer interfaces (BCI) which may allow overtly unresponsive patients to get in touch with their environment. Although underlying brain injuries seriously affect the dynamics of brain activity, studies have demonstrated that some patients in UWS and MCS are capable of following commands and executing mental tasks such as motor imagery. This has been found in blood-oxygen-level-dependent (BOLD) responses from fMRI (e.g., Monti et al., 2010; Bardin et al., 2012; Stender et al., 2014; Bodien et al., 2017) or EEG spectral power analysis (e.g., Goldfine et al., 2011; Höller et al., 2013). Likewise, attention-related responses to specific events or stimuli that elicit event-related potentials (ERPs) in the EEG (e.g., Kotchoubey et al., 2005; Lulé et al., 2013; Spataro et al., 2018) have been identified. These findings suggest that some patients can be aware of their surroundings, but there is high interindividual (and perhaps also intraindividual) variability. Not all of the patients show reliably detectable brain responses in a given paradigm: Factors like disease duration and traumatic pathology are also related to performance (Kotchoubey et al., 2005). Since fMRI, PET and MEG are limited in their clinical use, EEG is a feasible alternative for bedside examinations and also for the detection of awareness, because ERPs and oscillations in EEG recordings have been suggested as relevant markers of consciousness that can predict the outcome of patients with DoC (Fischer et al., 2004; Kotchoubey, 2005; Luauté et al., 2005; Chennu et al., 2017; Steppacher et al., 2020).

EEG oscillations can be divided into several frequency bands with different spectral boundaries such as delta (<4 Hz), theta (4–8 Hz), alpha (8–12 Hz), beta (12–30 Hz) and gamma (>30 Hz). Alpha is the dominant frequency band in the EEG of healthy adults (Klimesch, 1999) with an active role in information processing (Klimesch, 2012). In DoC-patients, the relative power of these frequency bands is highly abnormal, typically with decreased power in the alpha and increased power in the delta band. Relative delta band power is higher in UWS than in MCS patients, whereas alpha power is higher in MCS compared to UWS patients (Lehembre et al., 2012a).

In healthy people, pre-stimulus oscillatory activity in cognitive tasks has been shown to affect conscious perception in the auditory, visual and somatosensory modality (Linkenkaer-Hansen et al., 2004; Henry and Obleser, 2012; Weisz et al., 2014; Benwell et al., 2017). Especially the power and phase of the alpha-frequency band can predict subsequent conscious perception. Multiple studies showed that pre-stimulus activity affects post-stimulus ERPs, especially regarding the P300 (Jasiukaitis and Hakerem, 1988; Haig and Gordon, 1998; Ergenoglu et al., 2004; Mathewson et al., 2009; Roberts et al., 2014) which has often been linked to conscious stimulus perception, but also the N100, an index of sensory registration (Intriligator and Polich, 1995). So far, studies relating pre-stimulus EEG activity with post-stimulus ERPs were conducted with healthy volunteers and also mostly in the visual or auditory modality. A study from Ai and Ro (2014) also suggested a relationship between pre-stimulus alpha power and the stimulus detection rate in a tactile perception task (Ai and Ro, 2014). P100 and N200 somatosensory ERPs (sERPs) amplitudes increased in perceived trials and had an inverted U-shaped relationship with the pre-stimulus alpha power, indicating the existence of an optimal level of pre-stimulus alpha power for tactile perception (Ai and Ro, 2014).

In the present study we examined how pre-stimulus EEG activity relates to post-stimulus somatosensory responses in individuals in DoC. Therefore we analyzed pre-stimulus oscillations and post-stimulus sERP-data in response to an active somatosensory oddball paradigm. Scalp potentials evoked by sensory stimuli in oddball paradigms are for example the tactile N140, which is functionally analogous to the auditory and visual N100 component, and the P300, a positive deflection in averaged EEG data. Because of the heterogeneity of brain lesions and side in our patients, we first analyzed the post-stimulus epochs of standard and deviant stimuli to see at which electrodes and in which time-interval differences between the stimuli were statistically significant. After that the latency of the amplitude maximum, the amplitude maximum and also the area under curve (AUC) of the post-stimulus sERPs in the significant time interval were determined and the relative power of frequency bands in the pre-stimulus epochs of deviants were computed. We tested individually for each patient if the relative power of pre-stimulus frequency bands was correlated with post-stimulus variables to assess if pre-stimulus oscillation frequencies can predict the post-stimulus outcome in different stimulation conditions.

## Patients and methods

### Patients

Fourteen DoC-patients (6 female) who were stationary housed at the care facility “Haus Elim MeH,” Bethel, in Bielefeld from June to July 2018 participated in this study. The data sample consists of 13 MCS and 1 UWS patients (mean age-at measurement 44.29 years). Patients have been assessed through the early functional abilities (EFA) scale (Heck et al., 2000). Informed consent was obtained either from relatives or legal representatives for each patient. The research was approved by the ethics committee of the German Psychological Association. The individual demographic data for all patients are reported in Table 1.

**Table 1:** Patients demographic information.

#	Sex	Age at m.d.	Clinical State	Duration of illness (years)	Etiology
01	F	29	MCS+	10	HBD
02	F	67	MCS–	9	CCT/HBD
03	M	27	MCS–	10	CCT
04	M	54	MCS–	11	CCT/HBD
05	M	47	UWS/MCS	15	HBD
06	F	63	MCS–	13	HBD
07	M	35	MCS+	17	HBD
08	F	54	MCS	5	TI
09	M	38	MCS	2	HBD
10	F	42	MCS	2	ICH
11	F	42	MCS+	4	HBD
12	M	22	MCS+	3	IS
13	M	46	MCS	13	HBD
14	M	54	UWS	1	CCT

F, female; M, male; M.d., measurement date; MCS, minimal conscious syndrome; UWS, unresponsive wakefulness syndrome; HBD, hypoxic brain damage; CCT, craniocerebral trauma; TI, thalamus infarct; ICH, intracerebral hemorrhage; IS, ischemic stroke.

### Experimental procedure

**Figure 1:**
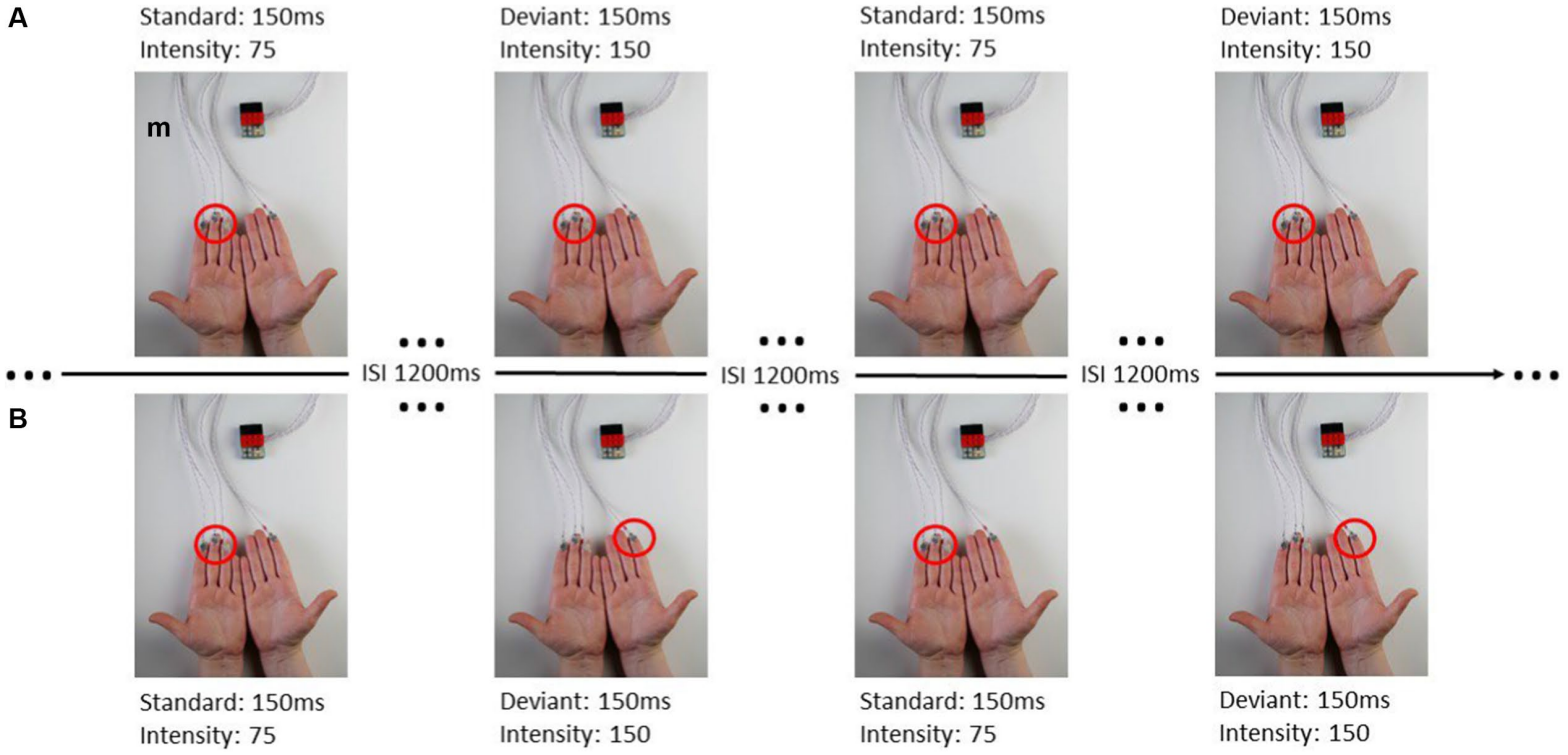
From Lindenbaum et al. (2021): **(A)** ipsilateral stimulation, **(B)** contralateral stimulation.

The experimental paradigm of this study is similar to the study by Lindenbaum et al. (2021). Vibrotactile stimuli were presented through the inhouse developed BRIX2 prototyping system (Zehe, 2018) with an extension-module consisting of cell-type-vibration motors (ERM; 10 mm × 3 mm; see Figure 1).

The intensity and duration of each vibration motor can be modified in the Arduino sketch-based firmware separately. The user can choose a value between 0 and 255 to adjust the amplitude of the vibration, where 0 is no vibration at all and 255 is the maximum vibration of the motors (100%). A fixed stimulus sequence with associated stimuli was created for each experimental run in OpenSesame (Mathôt et al., 2012). When stimulating body parts with neighboring cortical representation within one hemisphere, these representations interact, reflecting intra-hemispheric interference (Lindenbaum et al., 2021). This interference leads to decreased post-stimulus brain responses in the ipsilateral condition, whereas the contralateral stimulation requires a more complex interhemispheric connectivity. Therefor we defined four blocks of where and how patients were stimulated (see Table 2; Figure 1) including two ipsilateral and contralateral conditions. Each block consisted of 200 standard and 40 deviant stimuli. In the ipsilateral stimulation condition, three motors for standard and deviant presentation were always attached to the index, middle, and ring finger on one body site (left or right). In addition to the three motors (e.g., left fingers) for standard presentation a single motor was attached at the index finger (e.g., right index) for deviant presentation in the contralateral condition. The motors were attached to the fingers using adhesive plasters. Patients sat in a wheelchair or lay in their nursing bed and were instructed to focus on the deviant stimuli and count their occurrences.

**Table 2:** Experimental setup and spots of stimulus presentation.

Laterality of stimuli presentation	Standard stimuli	Deviant stimuli
Ipsilateral	Left fingers	Left fingers
Ipsilateral	Right fingers	Right fingers
Contralateral	Left fingers	Right index finger
Contralateral	Right fingers	Left index finger

### Stimuli

The characteristics and discriminability of stimuli presented in this study were previously tested with healthy subjects (Lindenbaum et al., 2021). Standard and deviant stimulus differed in their vibration-intensity but not in the duration of vibration. The duration of stimulus presentation was 150 ms. Standard stimuli had a peak frequency of 92.5 Hz and an intensity value of 75 (29.41% of the maximum vibration, magnitude 0.33 g) the deviant stimulus had a peak frequency of 175 Hz and an intensity value of 150 (58.85% of the maximum vibration, magnitude 0.8 g). The ratio of standard to deviant presentation was 5:1 and no two deviant stimuli followed consecutively. The inter-stimulus-interval (ISI) was 1,200 ms.

### Electroencephalography recording and preprocessing

Electroencephalography signals were recorded using a BioSemi system with 32 active electrodes,^1^ Cz as the recording reference and a sampling rate of 2,048 Hz. Off-line, data were down-sampled to 1,024 Hz and re-referenced to the average reference.

For all types of analyses, EEG-Data were pre-processed in BESA^®^ Research 6.0 using the automatic artifact rejection.

### Preprocessing for single-subject-sERP-analyses

The EEG-Data were pre-processed using a high-pass filter of 0.30 Hz (forward) and a low-pass filter of 15 Hz (zero-phase). We segmented the Data into epochs from 100 ms before stimulus (baseline) onset to 800 ms after Stimulus onset. Artifact-free epochs were submitted to single subject analyses in EMEGS (Peyk et al., 2011).

### Preprocessing for Fast Fourier Transformation

Artefact-free trials corresponding to deviant stimulation were segmented into epochs from 600 ms before stimulus to stimulus-onset (0 ms). The unfiltered data-epochs were then exported as BESA-text-files and converted to .txt-files using EMEGS (Peyk et al., 2011). The deviant epochs used for FFT-analysis were the same as in the single-subject-sERP-analyses.

## Data analyses

### Single-subject-sERP-analyses

The single-subject-analysis consisted of cluster-based permutation tests for each participant and condition. For each time-point and electrode, a two-sample *t*-test comparing the cortical response to the standard and deviant vibrotactile stimuli was calculated and compared against a distribution of 1,000 permutations and a cluster mass test (Maris and Oostenveld, 2007) as implemented in EMEGS (Peyk et al., 2011). This was done for two different pre-defined time-epochs, in which sERPs can typically expected (N140: 0–250 ms, P300: 250–750 ms) as well as across the entire epoch (0–800 ms) with all electrodes. The entire epoch of the whole post-stimulus interval (0–800 ms) was also included to test for any atypical processing differences between standards and deviants. T-tests were performed in EMEGS (Peyk et al., 2011). With the t-values of the t-test the cluster mass test with the three different time-epochs with all electrodes was computed. The significance criterium of the cluster mass test was set to *p* < 0.05 and values of significant time intervals with significant channel groups were saved for further analyses.

We then averaged the standard epochs for every patient and condition and subtracted the average standard epoch from every related single-trial deviant epoch. The difference epochs were then averaged across significant channel groups in the respective significant time interval. *Via* in-house python-based software the most positive peak (maximum), relating to the post-stimulus P300, and its latency was identified in the significant averaged difference epochs and the area under the sERP curve (AUC) was calculated.

### FFT

In an in-house python-based script the unfiltered single-trial-files of pre-stimulus deviant-epochs were averaged across significant post-stimulus electrode clusters from the single-subject-analysis. Hereafter, the averaged data was filtered with a sixth order 100 Hz digital butterworth low-pass filter [cut-off frequency was normalized by dividing by half of the sampling frequency (Nyquist-frequency)]. A 50 Hz notch filter was also applied to filter out AC line noise. Filtered data epochs were then windowed using a Hanning taper and symmetrically zero-padded at both ends to 1,024 timepoints (1 s of data). After that the one-dimensional discrete Fourier Transformation for real input was calculated using the python library NumPy and the relative power of frequency bands was computed. The frequency bands and the defined spectral boundaries are listed in Table 3.

**Table 3:** Frequency band and the defined spectral boundaries.

Frequency band	Spectral boundaries
Delta	0–4 Hz
Theta	>4–8 Hz
Alpha	>8–12 Hz
Beta	>12–30 Hz
Gamma	>30–100 Hz

### Statistical analysis of relationship between pre-stimulus power and post-stimulus brain response

For statistical analysis of the relationship between pre-stimulus frequency power and post-stimulus ERP parameters a correlation matrix was computed for every subject, condition, and time interval separately. Therefore, relative power of frequency bands in the pre-stimulus interval, the maximum and latency of the post-stimulus difference amplitude, and the AUC of the post-stimulus significant time interval for every deviant trial were used for the analysis. The correlation coefficients are reported as Spearman’s rho. Given that a total of 15 correlations were calculated for each subject and condition, it is expected that some of them may be chance correlations.

## Results

### Single-subject-sERP-analysis

**Figure 2:**
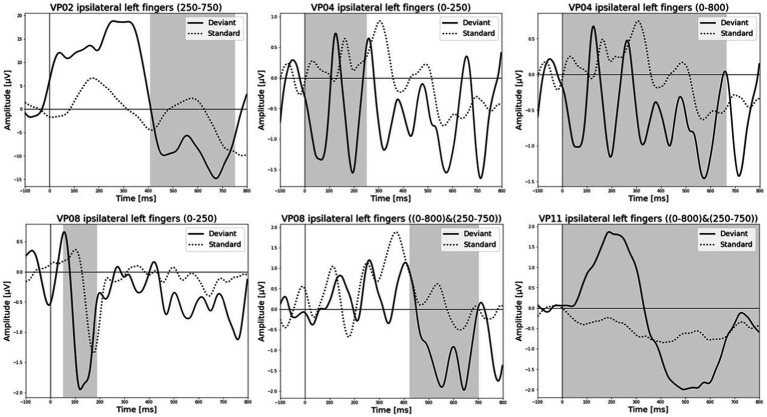
Grand-averaged event-related potential (ERP) waveforms of significant electrode cluster in response to the ipsilateral stimulation at the left fingers. The grey background shows the significant time-interval.

**Figure 3:**
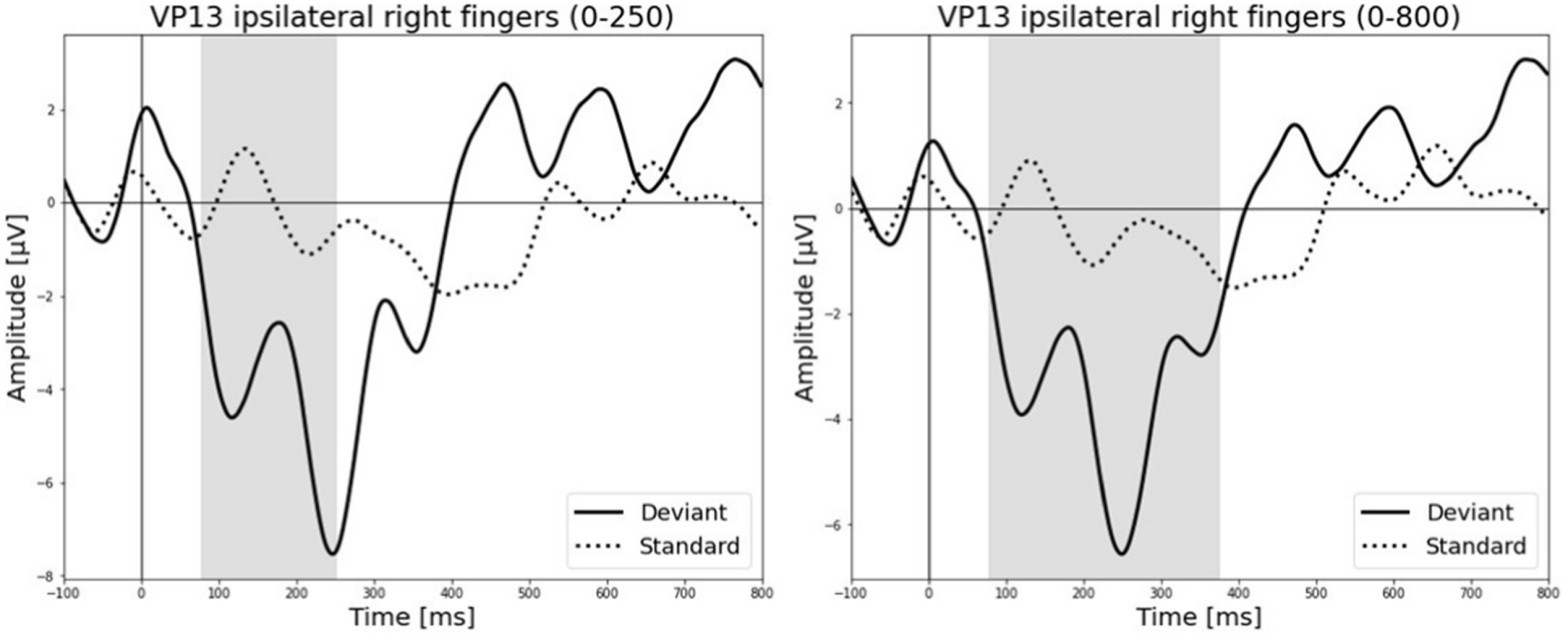
Grand-averaged ERP waveforms of significant electrode cluster in response to the ipsilateral stimulation at the right fingers. The grey background shows the significant time-interval.

**Figure 4:**
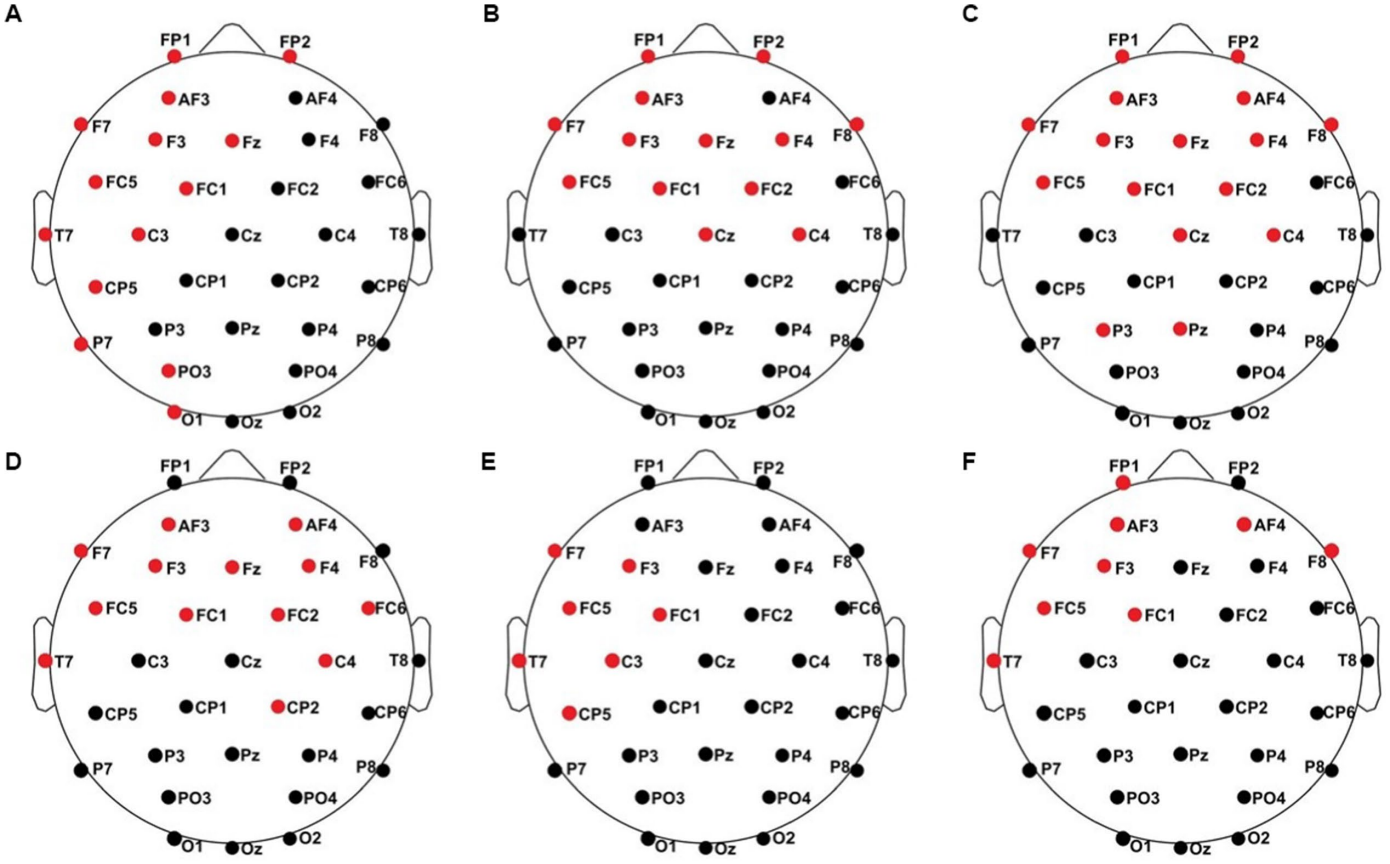
Significant electrode cluster (red) in response to the ipsilateral stimulation at the left fingers. **(A)** #02 (250–750 ms), **(B)** #04 (0–250 ms), **(C)** #04 (0–800 ms), **(D)** #08 (0–250 ms), **(E)** #08 (0–800 ms and 250–750 ms), **(F)** #11 (0–800 ms and 250–750 ms).

**Figure 5:**
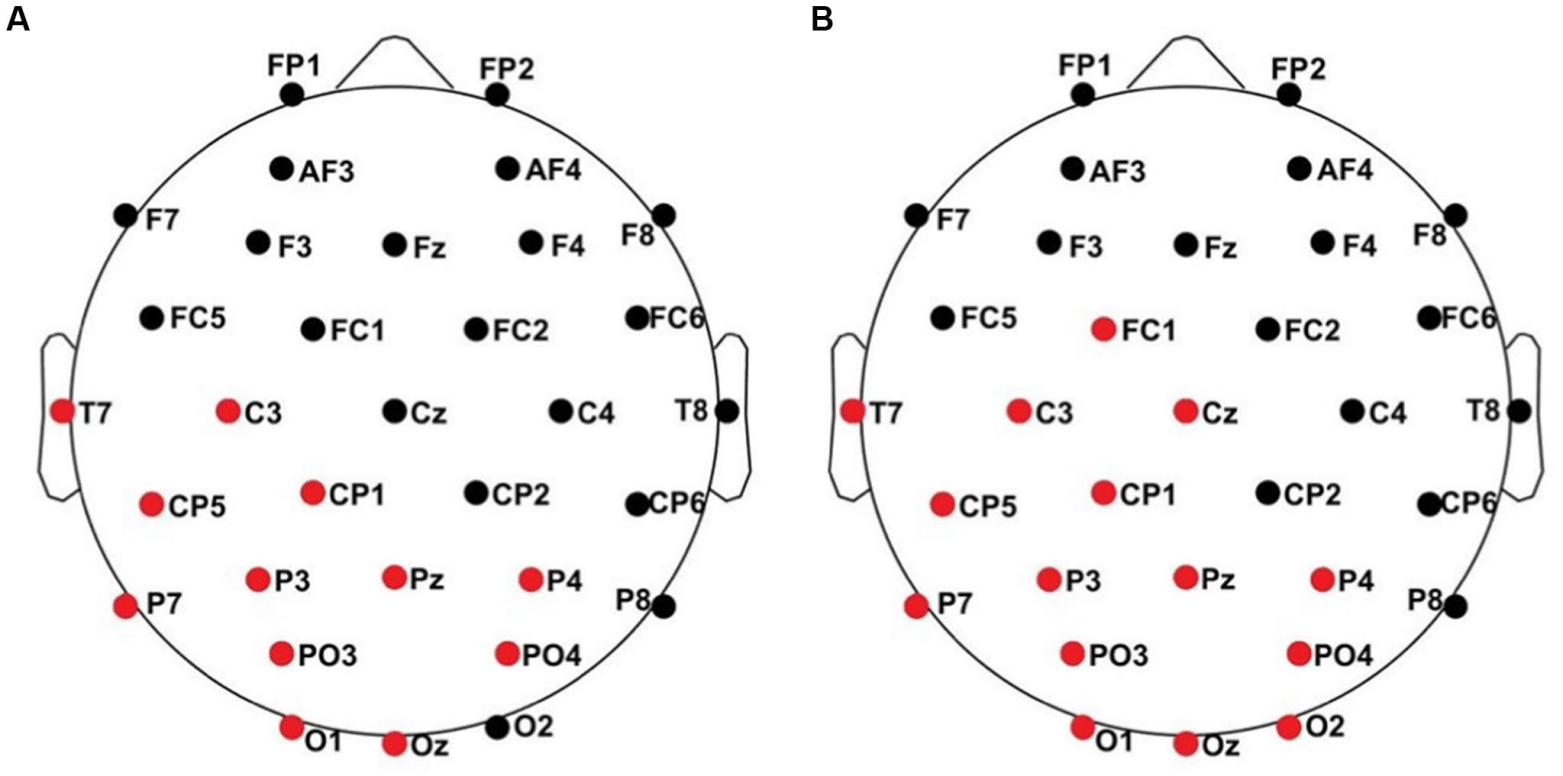
Significant electrode cluster (red) in response to the ipsilateral stimulation at the right fingers. **(A)** #13 (0–250 ms), **(B)** #13 (0–800 ms).

**Figure 6:**
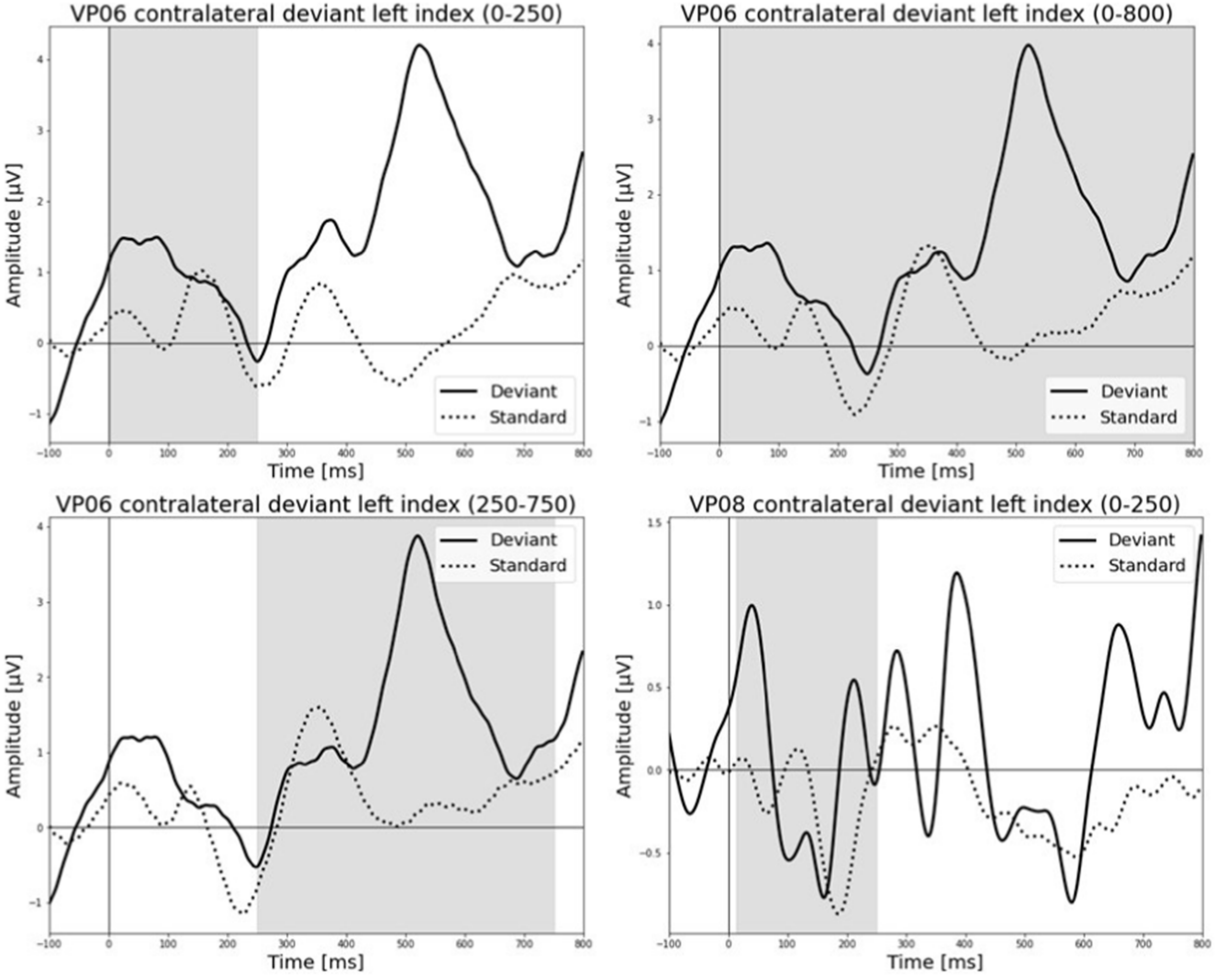
Grand-averaged ERP waveforms of significant electrode cluster in response to the contralateral stimulation with the deviant at the left index finger. The grey background shows the significant time-interval.

**Figure 7:**
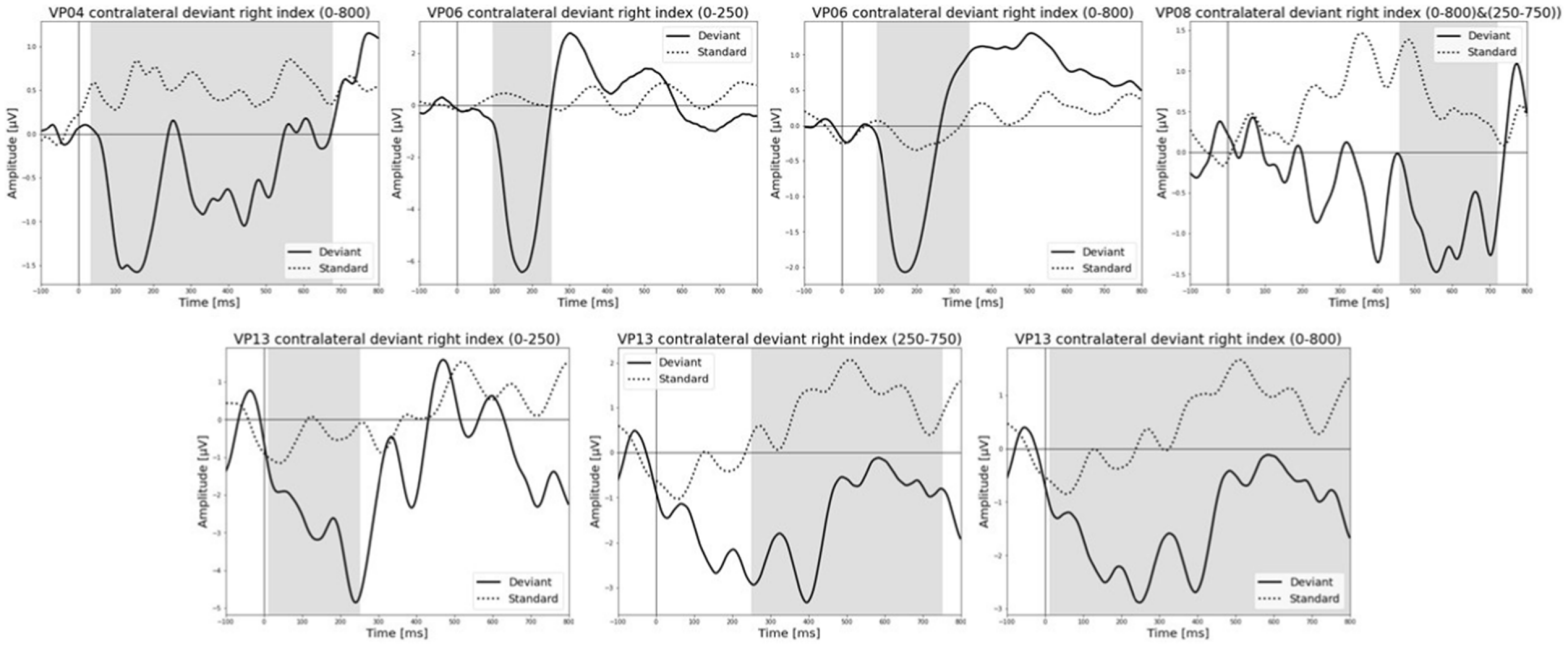
Grand-averaged ERP waveforms of significant electrode cluster in response to the contralateral stimulation with the deviant at the right index. The grey background shows the significant time-interval.

**Figure 8:**
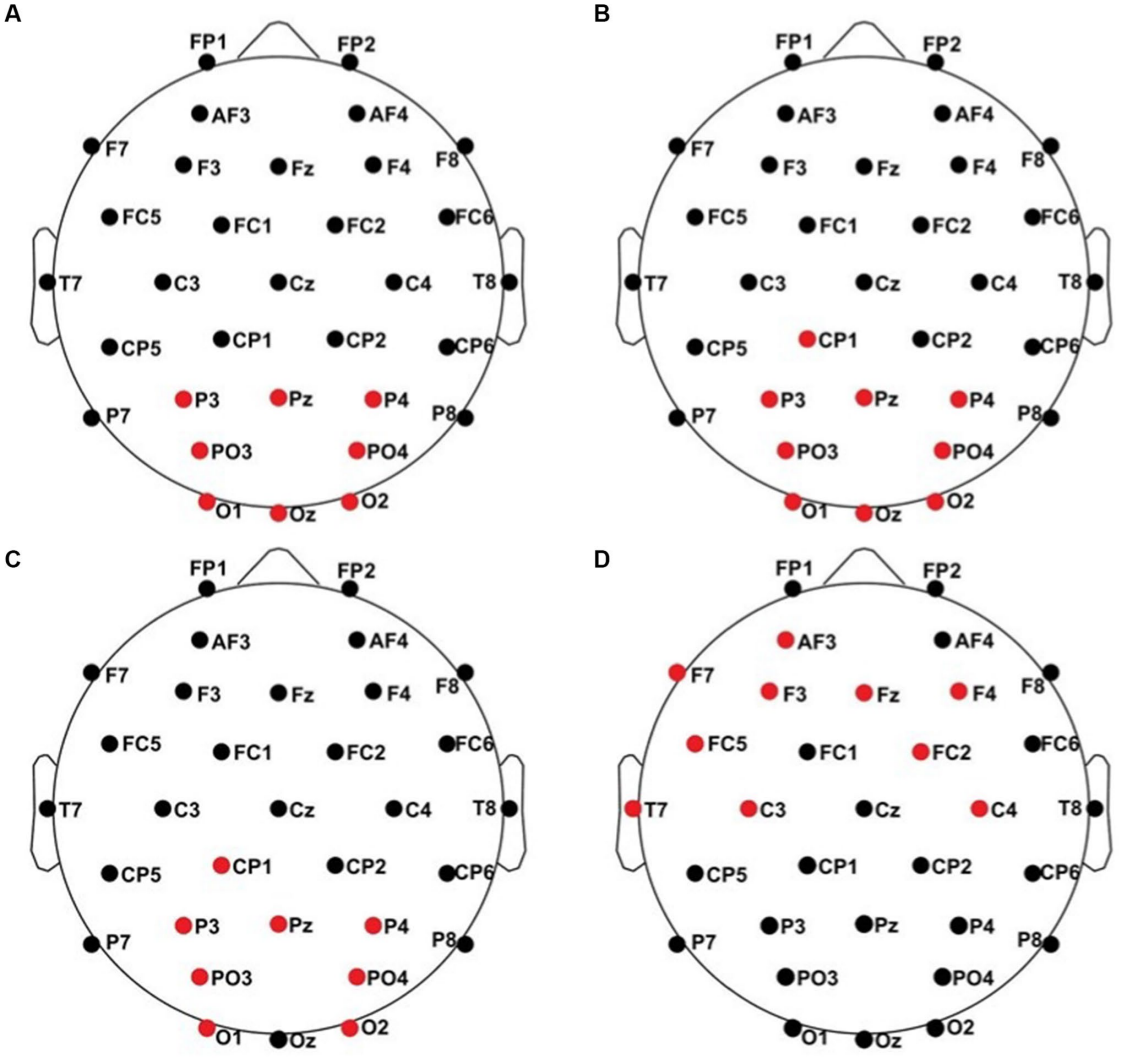
Significant electrode cluster (red) in response to the contralateral stimulation with the deviant at the left index finger. **(A)** #06 (0–250 ms), **(B)** #06 (0–800 ms), **(C)** #06 (250–750 ms), **(D)** #08 (0–250 ms).

**Figure 9:**
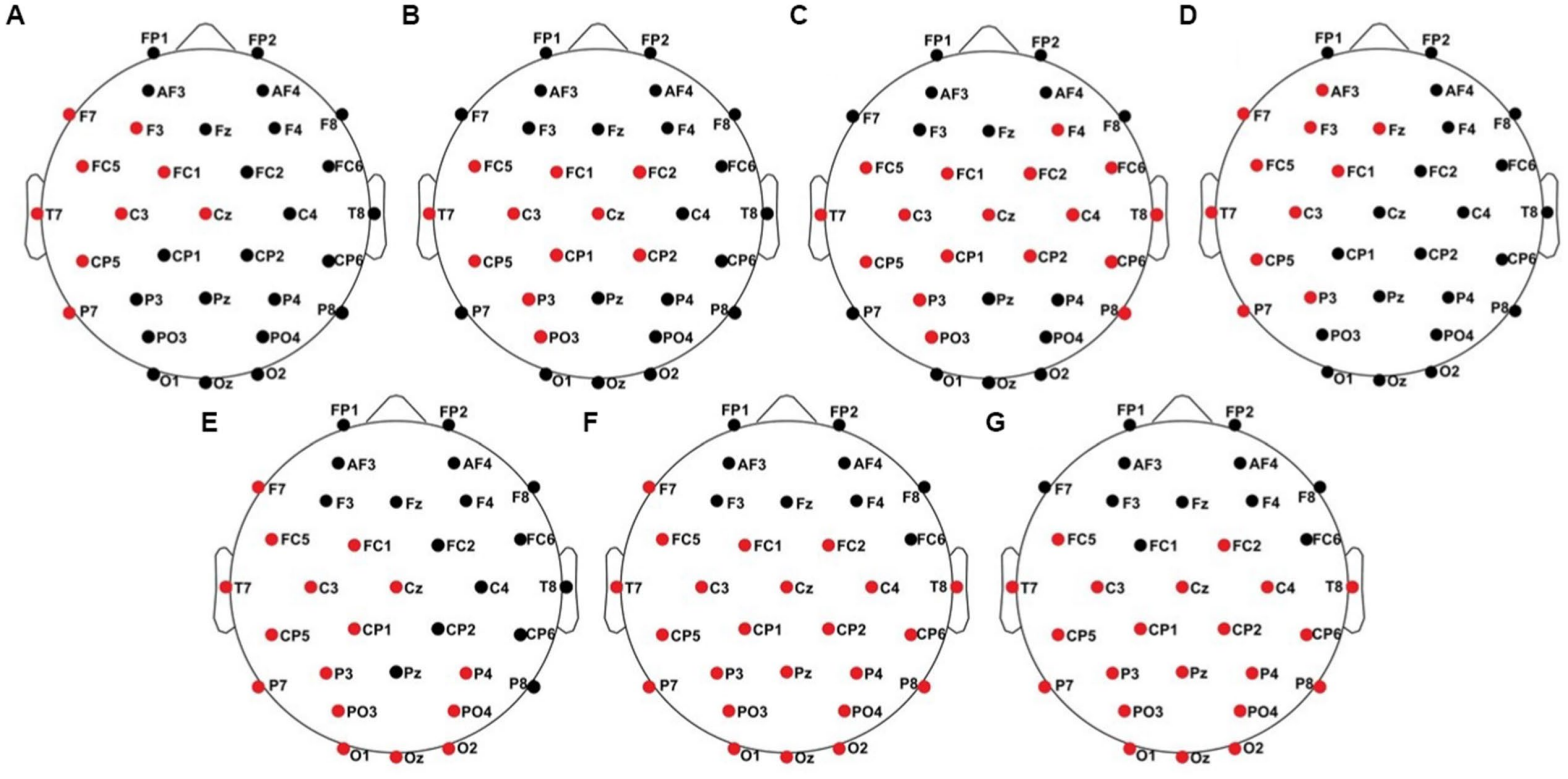
Significant electrode cluster (red) in response to the contralateral stimulation with the deviant at the right index finger. **(A)** #04 (0–800 ms), **(B)** #06 (0–250 ms), **(C)** #06 (0–800 ms), **(D)** #08 (0–800 ms and 250–750 ms), **(E)** #13 (0–250 ms), **(F)** #13 (0–800 ms), **(G)** #13 (250–750 ms).

All results of the single-subject-sERP-analysis are listed in Table 4. In six out of 14 subjects time intervals with significant differences between brain responses to standard and deviant stimulation were found and the data were suitable for further analyses. Ipsilateral deviant stimulation at the left fingers and the contralateral deviant stimulation at the right index finger most often led to significant differences in brain responses. Two subjects had significant intervals in only one stimulation condition whereas three subjects had significant intervals in two conditions and one subject had significant intervals in three conditions. The results of the ipsilateral stimulation at the fingers are shown in Figures 2, 3 with significant electrode clusters in Figures 4, 5. The results of the contralateral stimulation are depicted in Figures 6, 7 and the significant cluster to contralateral stimulations are shown in Figures 8, 9. The grey background in the waveform illustrations displays the time interval in which the differences of deviant and standard stimuli were significant (*p* < 0.05). Please note that waveforms of different time intervals in the same condition can differ, because they are based on different significant electrode clusters. Subjects 04 and 08 showed very jagged ERP response time-courses to especially the ipsilateral stimulation (see Figure 2) whereas other subjects showed better-defined courses of post-stimulus amplitudes.

**Table 4:** Results of single-subject-sERP-analysis.

Subject #	Condition	Time interval in analyses	Significant time interval (ms)	Cluster mass *p* Value	AUC Ø	Ø Max amplitude (μV)	Ø Latency max. amplitude (ms)
02	Ipsilateral deviant left fingers	250–750	408–750	*p* = 0.043	−2498.92	13.22	489.12
04	Ipsilateral deviant left fingers	0–250	4–250	*p* = 0.046	−210.60	3.49	150.76
		0–800	4–663	*p* = 0.038	−402.24	4.38	352.01
	Contralateral deviant right index	0–800	33–677	*p* = 0.029	−744.78	3.95	428.71
06	Contralateral deviant left index	0–250	0–250	*p* = 0.012	168.22	6.90	114.17
		0–800	0–800	*p* = 0.009	879.75	13.70	447.52
		250–750	250–750	*p* = 0.021	513.96	12.07	492.24
	Contralateral deviant right index	0–250	95–250	p = 0.01	−687.35	2.33	167.52
		0–800	95–339	*p* = 0.048	−138.32	1.86	273.52
08	Ipsilateral deviant left fingers	0–250	53–187	*p* = 0.032	−107.51	2.53	119.09
		0–800	424–700	*p* = 0.041	−314.23	5.59	557.54
		250–750	424–700	*p* = 0.022	−314.23	5.59	557.54
	Contralateral deviant left index	0–250	14–250	*p* = 0.042	64.23	4.43	114.53
	Contralateral deviant right index	0–800	459–719	*p* = 0.043	−430.50	3.14	615.93
		250–750	459–719	*p* = 0.017	−430.50	3.14	615.93
11	Ipsilateral deviant left fingers	0–800	0–800	*p* = 0.03	214.72	6.08	363.56
		250–750	250–750	*p* = 0.021	−119.62	4.16	495.02
13	Ipsilateral deviant right fingers	0–250	77–250	*p* = 0.024	−721.50	4.66	140.78
		0–800	77–375	*p* = 0.028	−992.75	5.91	222.34
	Contralateral deviant right index	0–250	12–250	*p* = 0.036	−569.23	5.64	112.68
		0–800	12–799	*p* = 0.009	−1513.94	6.94	379.77
		250–750	250–750	*p* = 0.009	−1262.13	6.31	261.19

Averaged AUC, maximum amplitude from the difference epochs (deviant-standard) and latency of the maximum in the significant time interval. Rows with grey background refer to the same time interval and/or electrode-cluster.

### FFT-analysis

**Figure 10:**
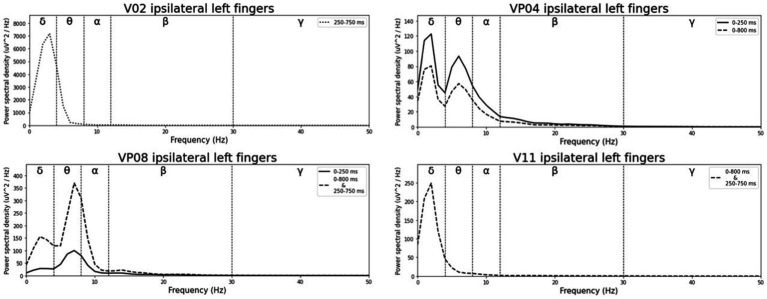
Averaged pre-stimulus frequency bands for ipsilateral stimulation at the left fingers.

**Figure 11:**
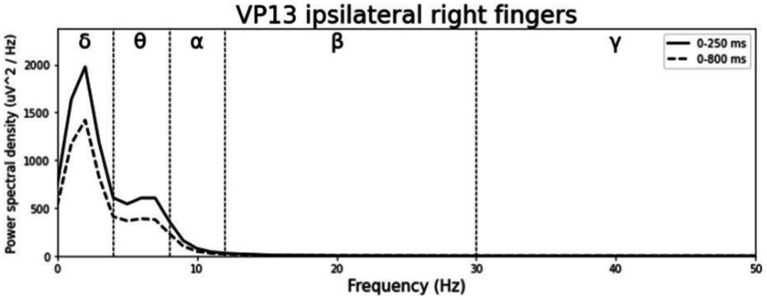
Averaged pre-stimulus frequency bands for ipsilateral stimulation at the right fingers.

**Figure 12:**
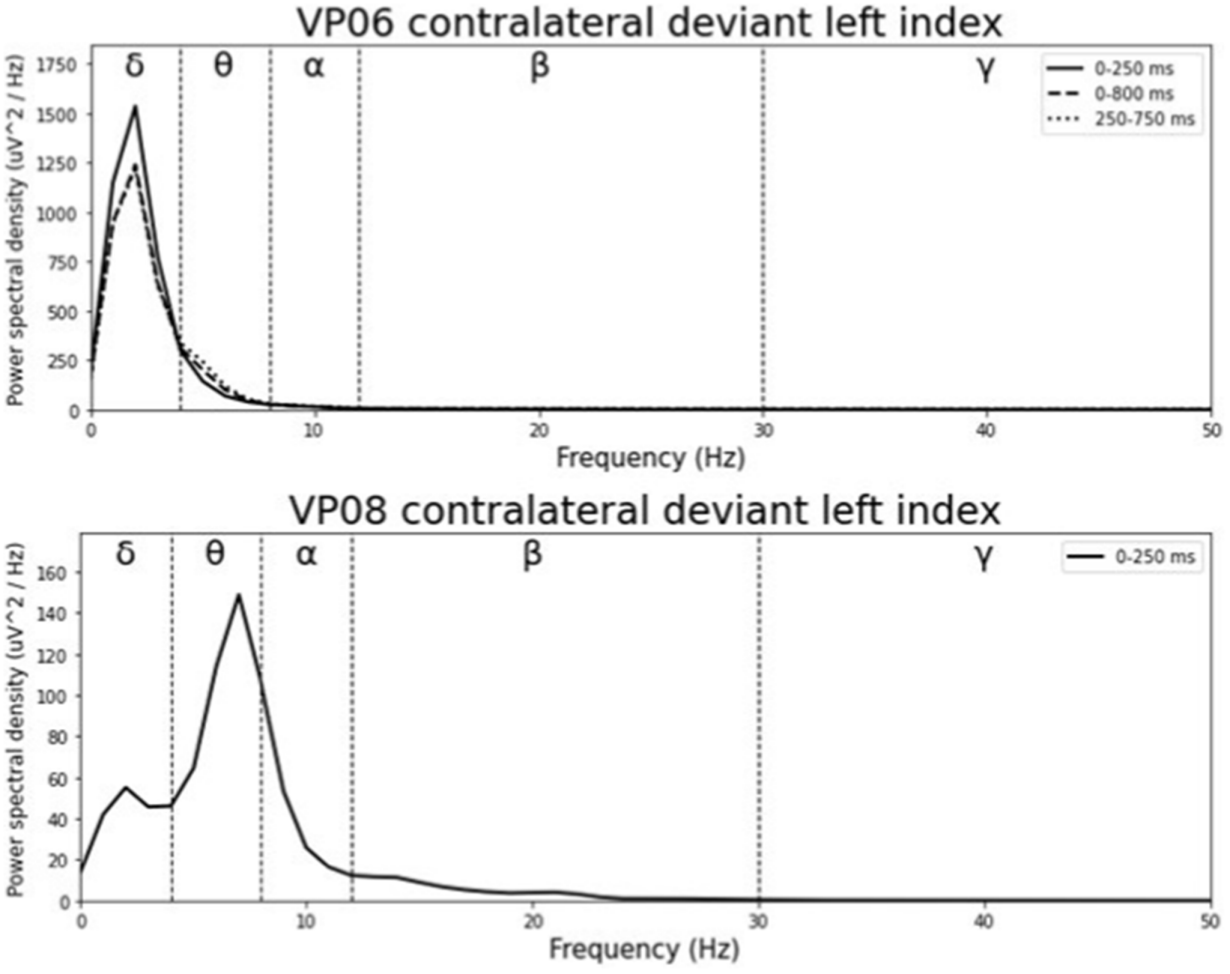
Averaged pre-stimulus frequency bands for contralateral stimulation with the deviant at the left index.

**Figure 13:**
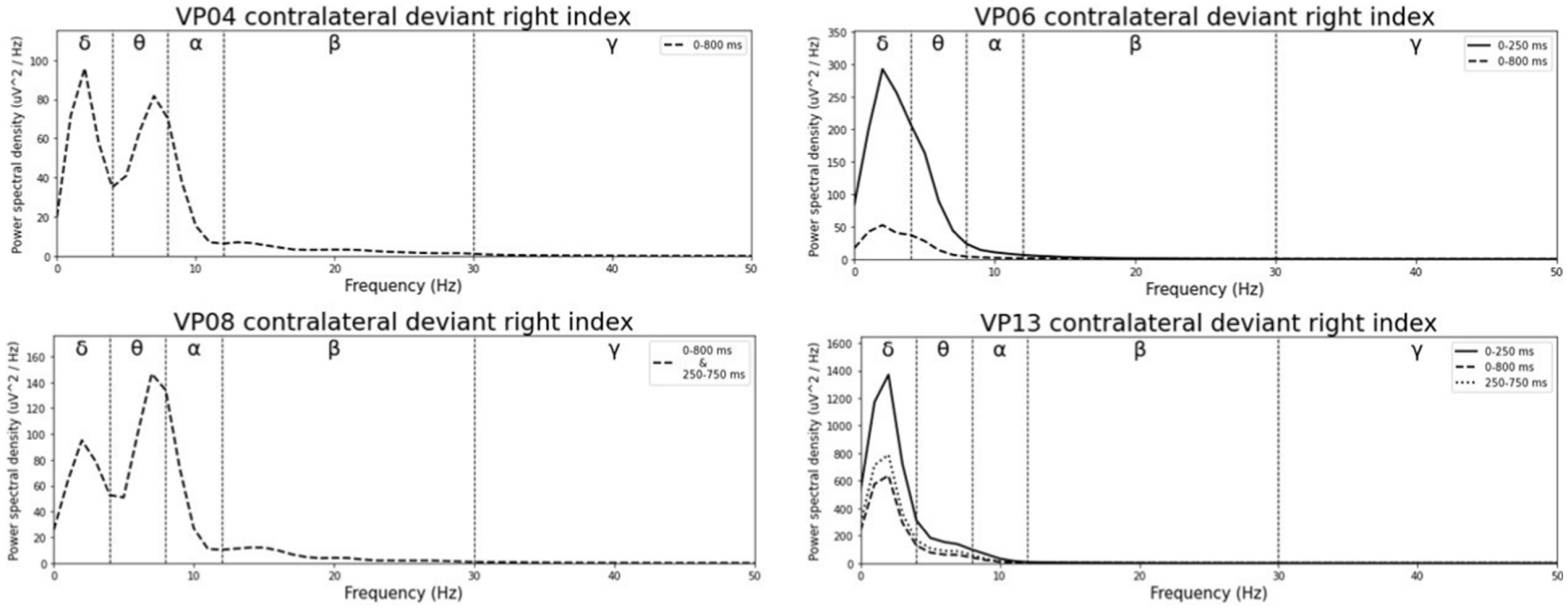
Averaged pre-stimulus frequency bands for contralateral stimulation with the deviant at the right index.

Averaged relative pre-stimulus frequency bands for deviant trials in patients who showed significant post-stimulus ERP differences between standard and deviants are listed in Table 5. The results of the ipsilateral stimulation at the fingers are shown in Figures 10, 11, results of the contralateral conditions are in Figures 12, 13. The power spectral densities (PSDs) of the significant electrode cluster in the significant time interval 0–250 ms are presented as black line, the time interval 0–800 ms as dashed black line and the 250–750 ms time interval as dotted black line. For the illustrations frequencies were cut at 50 Hz, because the gamma activity was so low in every subject that the PSD approaches zero. The percentage of frequency bands was the greatest in the delta range followed by the theta and alpha range for most of the subjects. One subject (#08) had a higher percentage of the theta than delta range whereas another subject (#04) had an almost similar percentage of delta and theta activity.

**Table 5:** Results of FFT-analysis.

Subject #	Condition	Significant time interval (ms)	Ø Delta (%)	Ø Theta (%)	Ø Alpha (%)	Ø Beta (%)	Ø Gamma (%)
02	Ipsilateral deviant left fingers	408–750	82.83	15.9	0.68	0.58	0.01
04	Ipsilateral deviant left fingers	4–250	38.6	34.59	13.87	9.94	2.78
		4–663	40.72	34.12	13.34	9.05	2.56
	Contralateral deviant right index	33–677	37.83	35.82	14.19	8.57	3.24
06	Contralateral deviant left index	0–250	88.48	9.38	1.32	0.74	0.08
		0–800	83.84	13.75	1.55	0.78	0.07
		250–750	81.62	15.77	1.73	0.81	0.07
	Contralateral deviant right index	95–250	64.77	29.7	3.31	2.01	0.2
		95–339	65.89	27.73	3.55	2.52	0.29
08	Ipsilateral deviant left fingers	53–187	17.31	51.57	19.52	11.28	0.30
		424–700	25.11	49.2	18.43	6.99	0.26
		424–700	25.11	49.2	18.43	6.99	0.26
	Contralateral deviant left index	14–250	21.38	50.59	18.41	9.19	0.40
	Contralateral deviant right index	459–719	29.32	41.22	18.72	9.15	1.48
		459–719	29.32	41.22	18.72	9.15	1.48
11	Ipsilateral deviant left fingers	0–800	86.41	8.63	2.05	2.02	0.82
		250–750	86.41	8.63	2.05	2.02	0.82
13	Ipsilateral deviant right fingers	77–250	66.36	27.11	5.42	1.07	0.04
		77–375	68.28	25.60	5.02	1.06	0.04
	Contralateral deviant right index	12–250	81.13	14.53	3.59	0.69	0.06
		12–799	82.94	13.65	2.69	0.65	0.07
		250–750	81.08	15.12	3.02	0.72	0.06

Averaged relative pre-stimulus frequency bands for deviants in significant electrode cluster. Rows with grey background means same time interval and/or electrode-cluster.

### Correlation-analysis

The results of the correlation analyses are listed in Tables 6–9. Five out of the six subjects showed significant results in the correlation analyses. The results of subject 02 were not significant in the correlation analysis. Subject 04 showed multiple moderate and weak correlations between pre-stimulus frequency bands and post-stimulus variables in the ipsilateral as well as in the contralateral stimulation (see Tables 6, 9), which might be due to the jagged course of post-stimulus response. Subject 06 showed positive correlations with the theta band, AUC and maximum amplitude (see Table 9) of post-stimulus intervals for the contralateral stimulation with the deviant presented at the right index finger. Subject 08 had positive and negative correlations in the ipsilateral and contralateral stimulation (see Tables 6, 8). In the ipsilateral stimulation at the left fingers, delta activity correlated positively with the AUC in the significant electrode- and time cluster 0–250 ms. In both, the 0–800 ms and 250–750 ms time-interval pre-stimulus alpha activity correlated positively with the latency of the maximum amplitude in post-stimulus time interval. In the contralateral stimulation with the deviant at the left index, alpha power correlated negatively with the AUC, with the AUC being more negative when the relative alpha power increased. Subject 11 showed significant correlations in both time intervals in the ipsilateral stimulation at the left fingers (see Table 6). In the time interval 0–800 ms, where alpha band power correlated negatively with the AUC and maximum amplitude post-stimulus. Maximum amplitudes decreased whereas relative alpha activity increased. Also, delta activity correlated positively with the maximum amplitude. In the time interval 250–750 ms the relative alpha band power correlated negatively with the maximum amplitude, with amplitudes decreasing whereas the relative alpha power increased. Subject 13 showed significant correlations in the ipsilateral as well as in the contralateral condition (see Tables 7, 9). In the ipsilateral stimulation at the right fingers alpha activity correlated with the maximum amplitude in both time intervals. In the contralateral condition the 250–750 ms time interval showed a significant positive correlation between the pre-stimulus beta activity and the AUC of post-stimulus amplitudes.

**Table 6:** Spearman’s rho of the correlation between pre-stimulus frequency band power and post-stimulus variables for the ipsilateral stimulation at the left fingers.

*#02 (250–750 ms)*	*α*	*β*	*γ*	*δ*	*θ*
AUC	−0.286	−0.571	−0.607	0.071	−0.071
Max. Amp.	−0.107	−0.464	−0.429	−0.107	0.107
Lat. Max. Amp.	0.394	0.374	0.374	−0.236	0.236
*#04 (0–250 ms)*	*α*	*β*	*γ*	*δ*	*θ*
AUC	0.363*	0.585***	0.330*	−0.344*	−0.018
Max. Amp.	0.218	0.359*	0.164	−0.344*	0.013
Lat. Max. Amp.	0.290	0.336*	0.163	−0.205	−0.154
*#04 (0–800 ms)*	*α*	*β*	*γ*	*δ*	*θ*
AUC	0.503**	0.499**	0.283	−0.293	0.025
Max. Amp.	0.377*	0.292	0.153	−0.194	−0.048
Lat. Max. Amp.	0.250	0.107	−0.010	−0.059	0.060
*#08 (0–250 ms)*	*α*	*β*	*γ*	*δ*	*θ*
AUC	0.133	0.010	0.010	0.342*	−0.281
Max. Amp.	0.118	−0.225	−0.175	−0.216	−0.028
Lat. Max. Amp.	−0.164	0.114	0.008	0.084	−0.206
#08 (0–800 and 250–750 ms)	*α*	*β*	*γ*	*δ*	*θ*
AUC	0.138	0.092	0.047	−0.012	−0.140
Max. Amp.	0.039	0.054	0.008	0.056	−0.033
Lat. Max. Amp.	0.334*	0.151	0.186	−0.114	−0.117
*#11 (0–800 ms)*	*α*	*β*	*γ*	*δ*	*θ*
AUC	−0.366*	−0.087	−0.151	0.219	−0.175
Max. Amp.	−0.390*	−0.303	−0.233	0.383*	−0.313
Lat. Max. Amp.	0.059	0.047	0.084	−0.053	−0.010
*#11 (250–750 ms)*	*α*	*β*	*γ*	*δ*	*θ*
AUC	−0.314	0.001	−0.105	0.170	−0.180
Max. Amp.	−0.345*	−0.221	−0.198	0.292	−0.224
Lat. Max. Amp.	0.104	0.147	0.010	−0.108	−0.061

^*^p < 0.05, ^**^p < 0.01, and ^***^p < 0.001. Significant correlations are highlighted in bold.

**Table 7:** Spearman’s rho of the correlation between pre-stimulus frequency band power and post-stimulus variables for the ipsilateral stimulation at the right fingers.

*#13 (0–250 ms)*	*α*	*β*	*γ*	*δ*	*θ*
AUC	0.368	0.277	0.116	−0.012	−0.112
Max. Amp.	0.445*	0.232	0.062	0.019	−0.186
Lat. Max. Amp.	−0.024	0.158	0.157	−0.230	0.252
*#13 (0–800 ms)*	*α*	*β*	*γ*	*δ*	*θ*
AUC	0.335	0.272	0.070	−0.048	−0.042
Max. Amp.	0.411*	0.295	0.036	−0.008	−0.109
Lat. Max. Amp.	−0.132	0.102	0.087	−0.158	0.211

^*^p < 0.05. Significant correlations are highlighted in bold.

**Table 8:** Spearman’s rho of the correlation between pre-stimulus frequency band power and post-stimulus variables for the contralateral stimulation with the deviant at the left index.

*#06 (0–250 ms)*	*α*	*β*	*γ*	*δ*	*θ*
AUC	−0.268	−0.111	−0.178	0.147	−0.113
Max. Amp.	−0.231	−0.104	−0.220	0.081	−0.012
Lat. Max. Amp.	−0.151	−0.119	−0.046	0.080	−0.041
*#06 (0–800 ms)*	*α*	*β*	*γ*	*δ*	*θ*
AUC	−0.281	−0.022	−0.116	0.080	−0.010
Max. Amp.	−0.177	−0.099	−0.127	0.020	0.004
Lat. Max. Amp.	−0.060	−0.075	0.043	−0.057	−0.003
*#06 (250–750 ms)*	*α*	*β*	*γ*	*δ*	*θ*
AUC	−0.197	−0.055	−0.080	0.035	0.046
Max. Amp.	−0.149	−0.156	−0.118	0.018	0.000
Lat. Max. Amp.	0.140	0.054	0.143	−0.093	0.017
*#08 (0–250 ms)*	*α*	*β*	*γ*	*δ*	*θ*
AUC	−0.353 *	0.122	0.023	−0.196	0.275
Max. Amp.	−0.058	0.267	0.041	−0.168	0.029
Lat. Max. Amp.	−0.213	−0.104	−0.012	0.091	0.125

^*^p < 0.05. Significant correlations are highlighted in bold.

**Table 9:** Spearman’s rho of the correlation between pre-stimulus frequency band power and post-stimulus variables for the contralateral stimulation with the deviant at the right index.

*#04 (0–800 ms)*	*α*	*β*	*γ*	*δ*	*θ*
AUC	−0.358*	−0.190	−0.265	0.346*	−0.333*
Max. Amp.	−0.377*	−0.354*	−0.374*	0.284	−0.141
Lat. Max. Amp.	−0.089	−0.101	−0.102	0.022	0.073
*#06 (0–250 ms)*	*α*	*β*	*γ*	*δ*	*θ*
AUC	−0.038	−0.014	−0.189	−0.050	0.041
Max. Amp.	−0.057	−0.037	−0.215	−0.251	0.266
Lat. Max. Amp.	0.078	−0.236	−0.143	−0.136	0.158
*#06 (0–800 ms)*	*α*	*β*	*γ*	*δ*	*θ*
AUC	−0.163	−0.003	−0.021	−0.296	0.349*
Max. Amp.	−0.089	0.055	−0.042	−0.321	0.370*
Lat. Max. Amp.	0.288	0.270	0.006	−0.089	0.026
*#08 (0–800 ms and 250–750 ms)*	*α*	*β*	*γ*	*δ*	*θ*
AUC	0.045	−0.074	−0.263	−0.059	0.178
Max. Amp.	0.158	−0.030	−0.234	0.063	−0.024
Lat. Max. Amp.	−0.066	0.019	−0.085	−0.274	0.186
*#13 (0–250 ms)*	*α*	*β*	*γ*	*δ*	*θ*
AUC	0.160	0.206	0.270	−0.248	0.231
Max. Amp.	−0.069	−0.004	0.036	−0.102	0.134
Lat. Max. Amp.	0.051	0.269	0.130	−0.151	0.199
*#13 (0–800 ms)*	*α*	*β*	*γ*	*δ*	*θ*
AUC	0.220	0.294	0.290	−0.237	0.220
Max. Amp.	0.080	0.126	0.143	−0.073	0.066
Lat. Max. Amp.	−0.098	0.072	0.065	−0.019	0.059
*#13 (250–750 ms)*	*α*	*β*	*γ*	*δ*	*θ*
AUC	0.247	0.394*	0.351	−0.267	0.238
Max. Amp.	0.050	0.234	0.171	−0.110	0.085
Lat. Max. Amp.	−0.134	−0.148	−0.205	0.190	−0.182

^*^p < 0.05. Significant correlations are highlighted in bold.

## Discussion

We investigated if pre-stimulus oscillation frequencies are related to post-stimulus cortical target differentiation in sERPs of patients in DoC. Fourteen DoC-patients participated in this study of whom six (42.86%) had significant differences between brain responses to standard and deviant stimulation in specific post-stimulus time intervals and electrode clusters. Thereof five subjects had multiple statistically significant correlations between pre-stimulus oscillations and post-stimulus variables. All of those were in MCS.

### Single-subject-sERP-analysis

Eight patients showed no significant differences between brain responses to standard and deviant stimuli (see Table 4). This finding is consistent with other studies showing that oddball responses, especially the P300, are less frequently detected in patients with DoC than healthy individuals (Gott et al., 1991; Signorino et al., 1997; Kane et al., 2000). All of our patients had enough artifact free trials for the analysis, so this outcome cannot be reduced to insufficient data or artefacts such as excessive motion, although involuntary head and/or body movements are very common in DoC patients. The absence of significant results in these patients does not necessarily indicate a general absence of significant differences in brain responses to certain stimuli. It could be the case that the stimulation paradigm used in this study is not effective for all our patients and that a different type of stimulation (e.g., auditory) would have led to significant differences in these patients.

In a study of Rousseau et al. (2008) four patients in the persistent vegetative state were stimulated in a tri-modal design (auditory, visual and somatosensory) to elicit evoked potentials (EPs). One patient showed EPs in the auditory and somatosensory, but not in the visual paradigm; another showed neither auditory nor somatosensory EPs, but, somewhat abnormal, visual EPs; the third patient showed somatosensory evoked potentials, but only in the right hemisphere and small visual evoked potentials, but auditory EPs were missing; and the last patient showed somatosensory and abnormal visual EPs, but the auditory evoked potentials were absent. These results show that not all modalities lead to brain responses in every patient and that the stimulation paradigms may need to be chosen individually for patients in DoC. The same applies to our stimulation paradigm. No patient showed significant results in all stimulation conditions but it appears that for some of them (e.g., patient 06) the contralateral stimulation leads to significant results whereas the ipsilateral presentation does not. Moreover, a certain presentation side of stimuli (right vs. left) can be more effective (e.g., patient 13).

Also, other factors like fatigue or fluctuations in arousal could have led to the inability to find significant results in several patients. It might be that the recording session was performed during a time of decreased level of activity due to arousal fluctuations and therefor with the incapability of the patient to maintain attentive and distinguish between the stimuli (Neumann and Kotchoubey, 2004).

It is challenging to visually identify the presence and absence of post-stimulus sERPs in patients 04 and 08 because the waveforms of post-stimulus interval were very jagged in the ipsi—as well as in the contralateral conditions (see Figures 2, 6, 7) but the statistical analyses revealed that the brain responses to deviant stimuli were significantly different from standard ones in specific time intervals and electrode clusters. On the other hand, the sERP curves of patient 06 even showed well-defined ERP components (N140 and P300) to deviant stimuli in the contralateral stimulation at the right index with significant time intervals in the specific time domain of the N140, despite a rather abnormal pre-stimulus power spectrum. Our results are quite variable, this could be due to the underlying injury, current (asleep) or general (diagnosis) clinical condition.

### FFT

Our results showed that in four of the six patients, in whom significant post-stimulus effects could be found, pre-stimulus relative power consisted predominantly of the delta frequency band (see Table 5; Figures 10–13). Patient 08 had a higher relative power of the theta than the delta band and patient 04 had an almost similar percentage of delta and theta activity. The order of the pre-stimulus relative power of frequency bands in these six DoC patients, ranked by percentage, was delta, theta, alpha, beta, and gamma.

Healthy awake adults show symmetric alpha rhythm at posterior electrodes at rest with a superimposed beta rhythm when they are attentive to their environment (Lehembre et al., 2012b). The theta rhythm is predominant when the individual is tired and the delta rhythm is predominant in deep sleep states (Lehembre et al., 2012b). The power of frequency bands is highly abnormal in DoC-patients with decreased power in the alpha and increased power in the delta band and also with differences in UWS compared to MCS patients (Lehembre et al., 2012a). Also, many abnormal patterns can be observed in the EEG of patients in DoC. Besides diffuse slowing activity continual focal polymorphic delta rhythm and epileptiform activity can be observed over damaged regions (Brenner, 2005). Our results are in line with previous findings (Nagata et al., 1989; Claassen et al., 2004; Fellinger et al., 2011) showing that the delta band power is predominant in DoC-patients.

### Correlation-analysis

Several studies have investigated the relationship between pre-stimulus oscillations and post-stimulus outcomes in healthy participants. Pre-stimulus activity, especially in the alpha band, can affect the early and late post-stimulus ERPs with regard to the amplitude maximum and its latency (Jasiukaitis and Hakerem, 1988; Intriligator and Polich 1995; Haig and Gordon, 1998; Ergenoglu et al., 2004; Mathewson et al., 2009; Roberts et al., 2014). Moreover, lower frequency bands in pre-stimulus epochs have been found to affect post-stimulus ERPs and evoked potentials (EPs), especially early ones such as N100 and N200 (Romani et al., 1988; Intriligator and Polich, 1995).

Five out of the six patients, in whom significant differences between brain responses to standard and deviant stimuli in the single-subject-sERP-analysis could be found, showed significant correlations between the relative power of frequency bands in the pre-stimulus interval and various indices of post-stimulus responses to deviant stimuli (see Tables 6–9). The pre-stimulus frequencies and post-stimulus variables of patient 02 did not correlate significantly. Patient 04 had multiple moderate and weak correlations in the ipsilateral as well as in the contralateral stimulation condition (see Tables 6, 9). It is quite challenging to interpret this outcome, but it might be reduced to the jagged course of post-stimulus reaction especially in the ipsilateral condition. Interestingly, this person had a relatively normal pre-stimulus frequency spectrum. The results to the contralateral stimulation at the right index of patient 06 are listed in Table 9. We found two positive correlations between the pre-stimulus theta band and post-stimulus AUC and maximum in the early post-stimulus time interval (95–339 ms) referring to the N140. Intriligator and Polich (1995) conducted a study to examine the relationship between EEG spectral power and post-stimulus auditory ERPs (aERPs) in healthy young adults. They found a negative correlation between the theta band and the size of the auditory N100. Also, results by Romani et al. (1988) showed a relationship between pre-stimulus low-frequency amplitudes and post-stimulus auditory EPs. N1–P2 amplitudes were lower and N1 latencies longer with higher pre-stimulus low-frequency spectral power. Although results are very atypical and variable in patients in DoC, we could show that the post-stimulus ERPs of patient 06 are affected by pre-stimulus oscillations in a similar way as in healthy subjects.

Patient 08 also showed very diffuse courses in the post-stimulus interval but with shorter time-windows, where responses to standards and deviants differed. This pattern might be easier to interpret. In the ipsilateral condition (see Table 6) pre-stimulus relative delta power on patient 08 correlated positively with the AUC of post-stimulus interval. The course of the mean post-stimulus amplitude is shown in Figure 2 with significant differences in the N140 time-interval (53–187 ms). The higher the relative power of delta band in the pre-stimulus epoch, the less negative the AUC. This result is also in line with previous findings in healthy subjects (Romani et al., 1988) and implicates that the relative power of pre-stimulus frequencies also affects post-stimulus ERPs, at least in some patients in DoC. A positive correlation between the relative power of alpha band and latency of maximal amplitude in the later post-stimulus time interval, approximately referring to a delayed P300 time range (424–700 ms), which is also in line with previous findings of delayed ERPs in DoC (Perrin et al., 2006), was found. Multiple studies showed that the pre-stimulus alpha activity affects the post-stimulus P300 ERP component (e.g., Jasiukaitis and Hakerem, 1988; Haig and Gordon, 1998; Ergenoglu et al., 2004). The results of the study of Intriligator and Polich (1995) also showed a correlation between the resting state alpha frequency band and the latency of the P300 aERP component. The P300 latency reflects the stimulus-processing time, e.g., the time a person needs to evaluate the stimulus (Kutas et al., 1977). This result is in line with multiple studies which showed that the detection of a visual stimuli is more likely with less alpha power in the pre-stimulus epoch (e.g., Ergenoglu et al., 2004; Mathewson et al., 2009; Roberts et al., 2014). For patient 08, the contralateral condition with the deviant at the left index, the pre-stimulus alpha power at the significant frontal electrode cluster correlated negatively with the AUC of post-stimulus N140 time-interval (14–250 ms). The higher the alpha power, the more negative the AUC. The N100 or in the somatosensory paradigm N140 reflects the discrimination process of stimuli. Roberts et al. (2014) found a relationship between pre-stimulus alpha-power and the N100 amplitude in a visual paradigm with more negative N100 amplitudes when pre-stimulus alpha power was low. Our findings suggest the opposite despite the fact that we computed the AUC of post-stimulus interval. The averaged N140 deflection of the patient (see Figure 6) does not show a single sharp peak instead it is more like a long trough, leading to a more negative AUC, which differs from the pattern in Roberts et al. (2014).

Patient 11 showed multiple significant correlations in the ipsilateral condition with stimuli presented at the left fingers (see Table 6) in two different time-intervals. We will focus on the later significant time-interval (250–750 ms), the expected time range of the P300, because the whole post-stimulus epoch (0–800 ms) was significant which makes the results of post-stimulus variables less meaningful. In the expected P300 interval the relative alpha band power correlated negatively with the maximal amplitude with more post-stimulus negative amplitudes when pre-stimulus alpha power was higher. Jasiukaitis and Hakerem (1988) results showed a positive relationship between pre-stimulus spectral power in the alpha band and amplitude of the P300 in an auditory paradigm. Our finding is in line with the finding of Jasiukaitis and Hakerem (1988). The visual inspection of sERPs in the post-stimulus epoch (see Figure 2) shows inverse curves to expected courses of a healthy subject but with time-intervals where brain-responses to the stimuli are significantly different. The negative correlation can be reduced to the inverse amplitude of the P300. This outcome is not unusual. ERPs in DoC are different from ERPs of healthy participants. They are smaller, delayed and sometimes their polarity is inversed (Perrin et al., 2006; Pokorny et al., 2013).

The results of patient 13 are listed in Table 7 for the ipsilateral condition with stimuli presented at the right fingers and in Table 9 for the contralateral condition with deviants presented at the right index. In the ipsilateral condition results showed a positive correlation between pre-stimulus relative alpha power and the maximal amplitude in both significant time-intervals (77–250 ms & 77–375 ms). The more relative power of the alpha-band the less negative post-stimulus N140 amplitudes. This finding is in line with the results of Roberts et al. (2014). They could show that, in a visual stimulation paradigm, the N100 amplitude increases with decreased pre-stimulus alpha power. Although the post-stimulus sERP curves are unusual, we could show that the post-stimulus N140 of this patient is affected by pre-stimulus oscillations in a similar way as has been previously reported in healthy subjects. In the contralateral condition results showed a positive correlation between pre-stimulus relative power of the beta band and the post-stimulus AUC in the significant interval (250–750 ms). The grand-average of post-stimulus ERPs (see Figure 7) shows a continuous negativity of the ERP but the more beta activity in pre-stimulus epoch the more positive the ERP becomes. Still, the maximum amplitudes did not correlate with beta so this outcome is hard to interpret. It is known that, at least in healthy people, pre-stimulus alpha modulates post-stimulus P300 in the auditory and visual paradigm whereas beta modulates earlier ERPs such as P1, N1, and P2 (De Blasio and Barry, 2013).

## Limitations and conclusion

We could show that pre-stimulus oscillations can be related to post-stimulus sERP also in patients in DoC. We found multiple correlations in the results of individuals which are in line with findings from studies with healthy volunteers. We only found significant differences between the standard and the deviant stimulus in six out of 14 subjects. This finding is consistent with other studies that have shown that not all patients in DoC who participated in an experiment had post-stimulus ERPs (e.g., Schoenle and Witzke, 2004; Kotchoubey et al., 2005). In most of the patients who had detectable sERPs, we were then also able to find a statistical relationship between pre-stimulus EEG power and post-stimulus deviant detection, although the direction did not always correspond to the direction previously described in healthy people. Given that we calculated 15 correlations for each patient, some chance correlations are to be expected. On the other hand, given the general changes in DoC patients’ EEG spectra, some atypical, but still functional brain dynamics are also likely. Furthermore, it is challenging to interpret the correlative outcomes for longer time-intervals (e.g., 0–800 ms) in post-stimulus epochs due to the conflation of early and late ERPs. Of note, at present all patients who showed detectable sERPs and subsequent correlations between pre- and post-stimulus brain activity were diagnosed as MCS, leaving open whether similar relationships might be present in UWS This could be tested in further studies and because our results are quite variable, larger groups of DoC patients would be desirable. So far, most ERP-studies in DoC are primarily conducted with auditory paradigms because patients in UWS and MCS cannot always control their eye movements or maintain eye-opening and fixate to a specific point in their visual field. The somatosensory modality might be a good alternative to the visual and even auditory paradigm as it is a basic sensory modality for interaction with both the physical and social environment and covers large cortical representation areas. Therefore, it should be considered for further investigations in patients in DoC. Our findings suggest that pre-stimulus oscillations do affect post-stimulus sensory and cognitive processing, albeit in a highly individual manner. Determining such individual relationships might help determine optimal stimulation windows for DoC patients, thereby increasing the likelihood of stimulation being processed and helping patients along the way to recovery.

## Data availability statement

The raw data supporting the conclusions of this article will be made available by the authors, without undue reservation.

## Ethics statement

The studies involving human participants were reviewed and approved by Ethik-Kommission der Universität Bielefeld. The patients/participants provided their written informed consent to participate in this study.

## Author contributions

LL and JK contributed to the conception and design of the study. LL, IS, and AM organized the database. LL performed the statistical analysis and wrote the manuscript. All authors contributed to the article and approved the submitted version.

## Funding

This work was supported by a grant from the German Federal Ministry of Education and Research (Förderkennzeichen 16SV7789K “NeuroCommTrainer”; Bundesministerium für Bildung und Forschung).

## Conflict of interest

The authors declare that the research was conducted in the absence of any commercial or financial relationships that could be construed as a potential conflict of interest.

## Publisher’s note

All claims expressed in this article are solely those of the authors and do not necessarily represent those of their affiliated organizations, or those of the publisher, the editors and the reviewers. Any product that may be evaluated in this article, or claim that may be made by its manufacturer, is not guaranteed or endorsed by the publisher.

## Footnotes

^1^ http://www.biosemi.com
